# Development and Application of Stereo Camera-Based Upper Extremity Workspace Evaluation in Patients with Neuromuscular Diseases

**DOI:** 10.1371/journal.pone.0045341

**Published:** 2012-09-17

**Authors:** Gregorij Kurillo, Jay J. Han, Richard T. Abresch, Alina Nicorici, Posu Yan, Ruzena Bajcsy

**Affiliations:** 1 University of California at Berkeley College of Engineering, Department of Electrical Engineering and Computer Science, Berkeley, California, United States of America; 2 University of California at Davis School of Medicine, Department of Physical Medicine and Rehabilitation, Sacramento, California, United States of America; University of South Australia, Australia

## Abstract

**Background:**

The concept of reachable workspace is closely tied to upper limb joint range of motion and functional capability. Currently, no practical and cost-effective methods are available in clinical and research settings to provide arm-function evaluation using an individual’s three-dimensional (3D) reachable workspace. A method to intuitively display and effectively analyze reachable workspace would not only complement traditional upper limb functional assessments, but also provide an innovative approach to quantify and monitor upper limb function.

**Methodology/Principal Findings:**

A simple stereo camera-based reachable workspace acquisition system combined with customized 3D workspace analysis algorithm was developed and compared against a sub-millimeter motion capture system. The stereo camera-based system was robust, with minimal loss of data points, and with the average hand trajectory error of about 40 mm, which resulted to ∼5% error of the total arm distance. As a proof-of-concept, a pilot study was undertaken with healthy individuals (*n* = 20) and a select group of patients with various neuromuscular diseases and varying degrees of shoulder girdle weakness (*n* = 9). The workspace envelope surface areas generated from the 3D hand trajectory captured by the stereo camera were compared. Normalization of acquired reachable workspace surface areas to the surface area of the unit hemi-sphere allowed comparison between subjects. The healthy group’s relative surface areas were 0.618±0.09 and 0.552±0.092 (right and left), while the surface areas for the individuals with neuromuscular diseases ranged from 0.03 and 0.09 (the most severely affected individual) to 0.62 and 0.50 (very mildly affected individual). Neuromuscular patients with severe arm weakness demonstrated movement largely limited to the ipsilateral lower quadrant of their reachable workspace.

**Conclusions/Significance:**

The findings indicate that the proposed stereo camera-based reachable workspace analysis system is capable of distinguishing individuals with varying degrees of proximal upper limb functional impairments.

## Introduction

A wide range of daily activities require unrestricted movement of the upper limb, primarily in the shoulder, to extend the reachability of the hand which is used to grasp, position or otherwise interact with various objects and environment. The concept of reachable/functional workspace is closely tied to range of motion (ROM) of the upper limb joints [1], [2]. In clinical practice, active range of motion assessment represents a quantitative method to evaluate movement and functional status of an impaired upper extremity [3]. Traditional ROM assessment can be obtained using goniometers or inclinometers [4], [5]. Although individual joint angles from such measures are helpful in evaluation of segmental function of the upper limb, it is often difficult to appreciate and readily visualize the overall functional capability of the upper limb based on a long list of joint angular ROM values, typically representing only the primary joint movements that are tested for each individual joint. In addition, appropriate application of these traditional ROM methods is operator-dependent, and present further sources of potential error, especially when dealing with a complex joint with multiple degrees-of-freedom as in a human shoulder joint. Furthermore, manual goniometry is often focused only on the extreme values of the ROM while ignoring the variability in joint mobility across the range.

A more in-depth characterization of the joint mobility can be obtained using motion capture systems with active or passive markers [6]–[9]. Although quantification and visualization of reachable/functional workspace is achievable through such a motion capture system, large costs and space requirements often limit their utility in clinical settings. Current motion capture systems are more suited to biomechanics or kinesthetic laboratories rather than doctor’s offices, therapy outfits, or even clinical research studies. Therefore, development of a simple, portable, and cost-effective reachable/functional workspace assessment of upper extremity that can be used practically in various clinical settings is desired, and represents the motivation for this study.

Recent advances in engineering, computing, and image processing techniques now allow stereo camera-based three-dimensional (3D) workspace analysis to be feasible. Currently, traditional upper-extremity evaluation including shoulder motion in clinical physiotherapy and physician/surgical practice has no 3D tool for an arm-function evaluation, which hampers a uniform, objective comparison. This is particularly true in the case of evaluating patients with various neuromuscular disorders, where majority of the weakness and upper limb dysfunction results from preferential involvement of the shoulder girdle muscles, and in turn resulting in impairment of reachable workspace. The envisioned system would use one simple stereo-camera setup with customized software program to acquire and characterize a patient’s upper limb 3D movement data; in essence, bringing some of the capabilities of a sophisticated motion capture laboratory to a clinic setting in a practical and cost-effective way.

In this paper, we present an innovative method to acquire and analyze quantitative measurement of reachable workspace of the upper extremity using a single stereo camera. In our setup, the camera captures a set of active LED markers attached to the patient’s skin to track the movement and positions of the hand, shoulder, and trunk. The collected motion data of hand trajectory is used to fit and segment a spherical surface which represents the reachable workspace envelope of the shoulder joint. We first evaluated and validated the developed stereo camera-based upper extremity workspace acquisition method against the traditional motion capture system in a controlled laboratory setting. As a proof-of-concept, we then evaluated the utility of the developed method in a clinical environment by collecting data on a group of healthy individuals and a group of patients with neuromuscular diseases and shoulder girdle weakness affecting their reachability to various degrees.

## Materials and Methods

### Ethics Statement

The study protocol was approved by the University of California Institutional Review Board for human protection and privacy. Each subject was first informed on the experimental procedure and written informed consent was obtained.

### Stereo System

To obtain position of markers in 3D space, at least two geometrically calibrated cameras with time synchronization are needed. For the measurements we have used BumbleBee2 camera (Point Grey Inc., Richmond Canada), which is a stereo camera with two imagers, each producing an image with the resolution of 1024×768 pixels at the frame rate of 20 FPS. The baseline of the camera, describing the distance between the two imagers, is 12 cm. The Bumblebee camera is compact and robust to mechanical disturbances. Once the camera is calibrated, it does not require re-calibration each time it is positioned on the tripod. The stereo camera was used in the clinical setting to track the location of different body landmarks marked with small LED markers.

### Tracking Application

Detection and labeling of markers from the images captured by the stereo camera are performed by the tracking algorithm described in this section. Data processing consists of the following steps: (1) marker detection, (2) marker tracking, (3) triangulation, and (4) workspace analysis. The marker detection from the images is performed via thresholding of the background-subtracted image, while searching for circular-shaped markers within specific radius range. The location of the marker center is determined by calculating the center of marker intensity with sub-pixel accuracy. The markers are classified based on the size, location and color. LEDs of different colors but of the same illumination properties were used to avoid marker-swapping when two markers are in close proximity of each other. The color information was added after initial tests on patients who did not exhibit typical smooth movements as those found in healthy individuals, which could be used to predict marker location in consecutive frames using conventional tracking methods. The smoothness assumption can be used to predict marker location in consecutive frames using conventional tracking methods.

**Figure 1:**
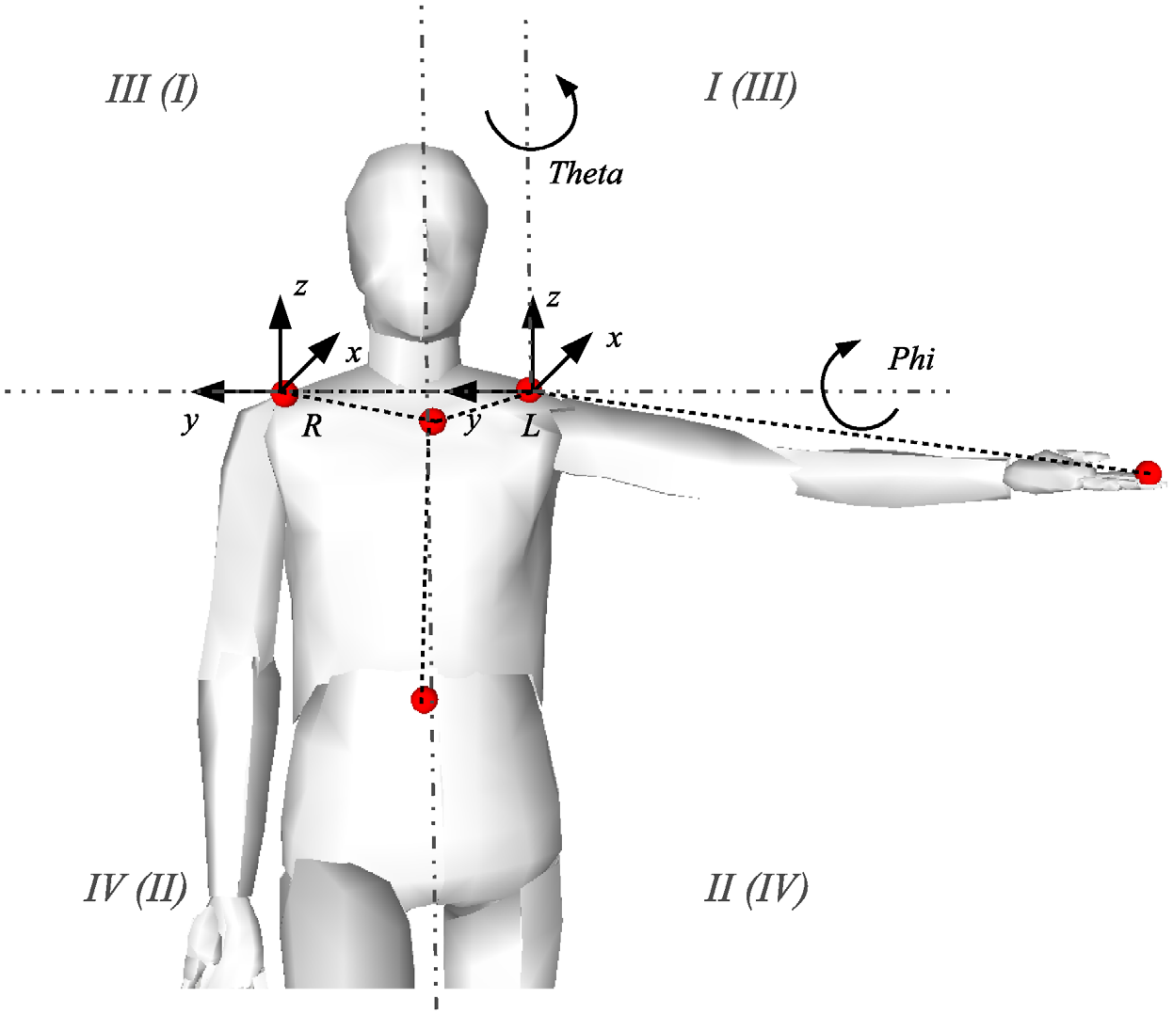
Application of markers on the body landmarks and the corresponding quadrant notation for the left and right arm (Roman numerals in the parentheses are for the right arm).

At the beginning of tracking, the subjects are positioned in a neutral position where all the markers are visible. Each marker is attached with a tracker consisting of the Kalman and condensation filtering to track marker position over time. At this point, the markers are also enumerated (Figure 1) to keep consistency over the measurements. For each tracker, possible marker candidates are determined from a combination of the Euclidian distance and color similarity between previous location of the marker and possible candidates. The color similarity is initialized in the first frame and updated every 10 frames of successful tracking. For all candidates, probabilities are determined and the marker with the highest probability is selected as the next position. The tracking algorithm combines the approaches as described in [10] and [11]. The algorithm can deal with short occlusions and marker path crossings. The location of the markers is determined in each view of the stereo camera independently. Since the marker enumeration is performed in the same way in both views, the matching can be done across the markers. Knowing the camera parameters (i.e. focal length, optical center, distortion coefficients, and baseline), we can apply triangulation algorithm to determine the 3D position of each marker with respect to the camera location.

### System Evaluation

The 3D measurements of the marker location were first evaluated in a controlled laboratory environment using the Impulse optical measurement system (PhaseSpace, San Leandro, CA). The motion capture system consists of eight cameras positioned in circular fashion which are able to accurately (with accuracy around 1 mm) track 3D location of active LED markers. The Impulse system uses frequency modulation to uniquely identify individual markers. Since the frequency modulation is large, the markers appear lit to the slower vision-based stereo cameras. The LEDs of the Impulse markers (Luxeon III, Phillips Lumiled) have identical properties as the LEDs used in our stereo system for the measurements in the clinical environment. The outputs of the stereo camera and motion capture system were temporarily aligned using Network Time Protocol (NTP) to synchronize the clocks of the acquisition computers. The stereo camera system was calibrated with the motion capture system coordinate system by a checkerboard rigged with three motion capture markers.

**Figure 2:**
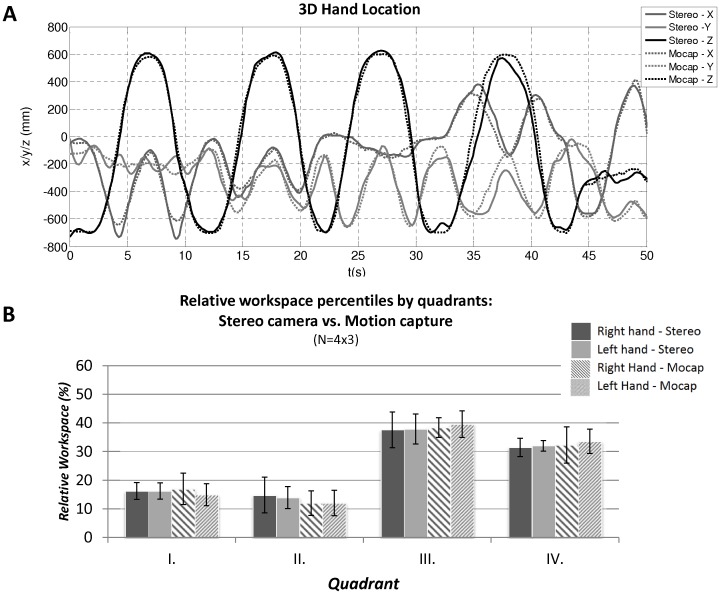
(A) Example of measured three-dimensional hand location as measured by the commercial motion capture system and stereo triangulation algorithm in a healthy individual. (B) Comparison of relative workspace percentiles measured by motion capture vs. stereo camera system for each respective quadrants in healthy individuals.

For the evaluation we have performed similar tests with four healthy subjects as planned for the study protocol. Tests with the motion capture system have shown that achievable accuracy is in the range of 2–4 cm for the z-range, however the accuracy varies across the measurement due to variability in the accuracy of the detection of marker centers in both views. The largest errors occur when markers are on the borderline of visibility (e.g. due to occlusions or large angles with respect to the camera direction). The tracking steps as described above are performed in real time, retaining the frame rate of about 15 frames per second (fps). The average hand trajectory error between the two systems after the transformation into the shoulder coordinate system was about 4 cm, which results to about 5% error of the total arm distance. Figure 2 (above) shows a typical output trajectory measured by the two systems after projection the hand marker into the shoulder coordinate system. We observed that the tracking error is the highest when the markers are close to occlusion points due to increased error in marker center detection. The average tracking error in four subjects over three trials each performing similar arm movement protocol was 33 mm with standard deviation of 12 mm.

### Experimental Procedure and Subjects

For motion data collection, we have tracked five markers applied to the upper torso and abdomen (suprasternal notch, acromion process, and umbilicus) and the tip of the middle finger. For the body markers we have use high luminance LEDs (Luxeon III, Phillips Lumiled). For the hand, a white light source supplied by a Maglite® pencil flashlight with diffuser removed to achieve highest level of visibility from any angle. The substitution of the marker color for the clinical experimental procedure did not affect the accuracy of the marker detection algorithm since the center of the marker is calculated from the intensity (grayscale) image.

Anthropometric measurements of arm length were obtained for each subject (distance between the acromion process LED and tip of middle finger where the white light marker was located). Subjects were seated in a chair, located about 2 m from the camera, with their arms at their sides (which was designated as the starting position, or the neutral position). The chairs had no arm supports or arm rests. The impaired individuals who were in a wheelchair performed the experiment from the wheelchair with the arm rests removed. A strap was applied below the axilla to minimize the movement of the trunk during the measurements. Markers were applied to the skin using simple velcro adhesive tapes. The subjects were then shown the study protocol movements by the study kinesiologist and instructed to mirror the movements. A standardized simple set of movements consisted of lifting the arm from the resting position to above the head while keeping the elbow extended, performing the same movement in vertical planes at around 0, 45, 90, 135 degrees. The second set of movements consisted of horizontal sweeps at the level of the umbilicus and shoulder. The entire sequence of movements was recorded together. The study protocol movements were simple to perform for the subjects and typically took less than 1 minute for the entire sequence of movements; yet, the shoulder underwent its full ROM (except for the extreme shoulder extension that is limited by the back of the chair). Each set of movements was repeated three times for left and right arm. Subjects were instructed to reach as far as they can while keeping the elbow straight. If they were unable to reach further, they were to return to the initial position and perform the next movement. During the measurements a kinesiologist demonstrated the movements in front of the subject to dictate the speed and order of movement segments, and if the subject leaned or trunk rotations were observed by the kinesiologist, the recording was repeated from the beginning. A total of 20 healthy individuals (12 female, 8 male; average age: 36.6±13.6 years) and 9 patients (all male but one, average age: 46.2±16.3 years) with various neuromuscular conditions participated in the study (Table 1).

**Table 1:** Patient data and corresponding results of the surface envelope assessment are presented as the average value over three trials.

					Arm	Quad				Surface	
	Age	Sex	Diagnosis	Side	Length	I	II	III	IV	Relative	Absolute
					(cm)	(%)	(%)	(%)	(%)	(−)	(m^2^)
P1	29	M	BMD	**Right**	71.6	2.3	0.3	49.9	47.5	0.42	1.36
				**Left**	71.0	0.0	0.0	52.2	47.8	0.21	0.67
P2	29	M	Pompe	**Right**	77.3	0.0	0.0	0.0	100.0	0.14	0.51
				**Left**	77.3	0.0	0.0	0.0	100.0	0.06	0.24
P3	49	M	BMD	**Right**	69.9	0.9	0.2	49.0	49.9	0.44	1.34
				**Left**	70.6	5.5	1.1	51.2	42.2	0.44	1.37
P4	55	M	BMD	**Right**	79.5	6.1	11.4	34.7	47.8	0.53	2.10
				**Left**	80.3	4.6	5.3	43.0	47.1	0.50	2.02
P5	47	M	BMD	**Right**	73.0	7.7	8.3	39.4	44.6	0.62	2.09
				**Left**	73.5	12.5	0.6	49.1	37.9	0.50	1.68
P6	54	M	BMD	**Right**	80.5	0.0	0.0	0.0	100.0	0.03	0.12
				**Left**	82.0	0.0	6.7	0.0	93.3	0.09	0.37
P7	49	M	FSHD	**Right**	84.0	0.0	0.0	0.2	99.8	0.08	0.37
				**Left**	84.5	0.0	1.4	0.0	98.7	0.08	0.35
P8	70	F	FSHD	**Right**	70.5	3.9	0.1	52.7	43.3	0.47	1.46
				**Left**	71.2	9.5	3.6	47.9	39.1	0.38	1.21
P9	13	M	DMD	**Right**	66.0	0.0	2.9	40.3	56.8	0.28	0.78
				**Left**	66.0	0.0	0.0	0.0	100.0	0.04	0.12

* BMD: Becker muscular dystrophy; DMD: Duchenne muscular dystrophy; FSHD: Facioscapulohumeral dystrophy; Pompe: Pompe disease (glycogen storage disease type II).

### Reachable Workspace Envelope Analysis

**Figure 3:**
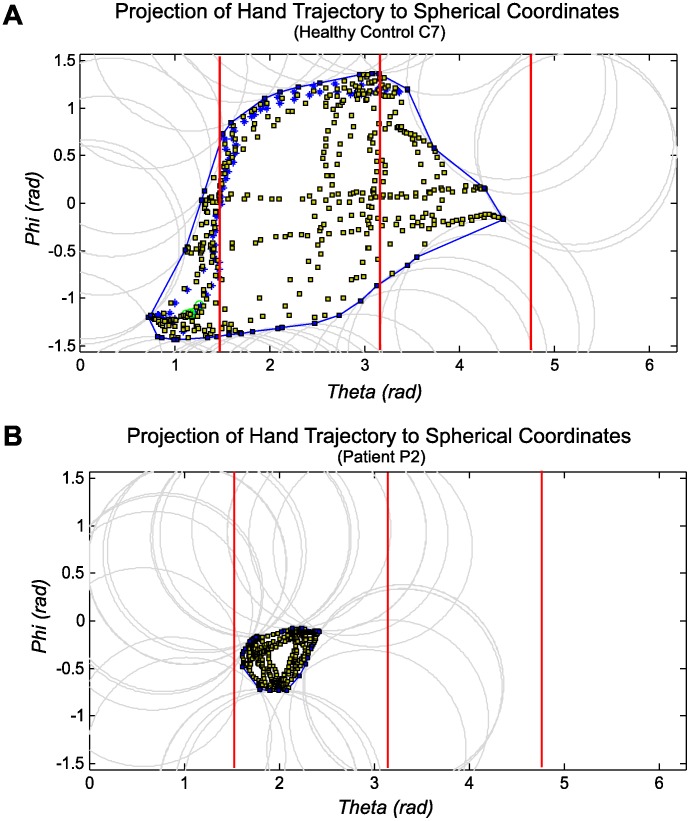
Hand trajectory is projected to spherical coordinates to obtain the outer boundaries of the concave bounding polygon using alpha shapes. The boundary is applied to cut out the 3D spherical surface of the reachable envelope. For a control subject (A), the surface area lies in the interval of [π/2, 3π/2], while the surface area obtained in the patient with Pompe disease (B) is significantly reduced.

The analysis of the workspace envelope was performed offline. The tracked 3D hand trajectory was first transformed into body-centric coordinate system defined by the four markers on the body. The data was filtered with 3^rd^ order Butterworth filter with the cut-off frequency of 10 Hz. Large outliers (i.e. spikes) due to triangulation error were removed using implementation of phase-space despiking method presented by Mori *et.al* [12]. In 3D space, the obtained hand trajectory can be interpreted as a point cloud where the points lie on a surface of the reachable envelope of the arm. To simplify the analysis we fitted a spherical surface similar as in [13] into the data points. Due to noise and the simplification of the shoulder joint, some of the points were offset from the surface, however, the errors were in the order of few centimeters. To obtain the boundaries of the surface, the data was first transformed into spherical coordinates by projecting the points close to the sphere onto the surface of the sphere and eliminating outlying points. Since the radius was fixed, the projected data was two-dimensional and parameterized with the corresponding vertical and horizontal angles. Figure 3 shows the projection of the 3D trajectory into the spherical coordinates with the corresponding boundary polygon. The parameterization follows the angle directions as shown in Figure 1. The boundary points were obtained using alpha shape [14]. Alpha shape consists of piece-wise linear curves which approximate a concave surface containing the set of points. The level of concavity is defined by the circumscribed circle defined along the convex boundary as shown in Figure 3 (circle radius was π/4). Figure 3 shows two example outputs from individuals with and without upper limb functional impairments. The spherical surface represented by small rectangular patches (i.e. quads) was segmented using the boundary curve of the alpha shape. The quads were culled depending whether their centers lie within the alpha shape in the spherical coordinates or not. Furthermore, we split the surface data into four quadrants corresponding to the coordinate system placed in the shoulder joint and defined by the standardized human body planes. The sagittal plane defined the left and right side of the workspace and the horizontal plane (at the level of the shoulder joint) defined the top and bottom part of the workspace. The quadrants are enumerated as shown in Figure 1. We calculated the reachable surface area for each of the quadrants and the summated total area, as well as the relative surface area. The relative surface values are reported as a percentage of the total surface area. The surface area was normalized with respect to surface area of a unit hemi-sphere (with radius 1.0) to be able to compare the results between subjects. The assessed relative surface area therefore lies between 0.0 and 1.0, where 1.0 represents reachable workspace envelope of the entire (frontal) hemi-sphere.

### Statistical Analysis

For the analysis of the average values of the parameters, we report arithmetic mean and the corresponding standard deviation interval. We have applied one-way analysis of variance to determine the significance of the measurement population differences between male and females controls. To analyze statistical significance of patient results, one sample t-test for the healthy subjects was used against a test value from a single patient. We considered *p*-values of 0.05 or less as statistically significant. The statistical analysis was performed with Matlab Statistical Toolbox.

## Results

Figure 3 shows an example of hand trajectories projected into spherical coordinates and fitted surface area as obtained in a healthy subject (C7) and an individual with Pompe disease with relatively severe phenotype of shoulder girdle muscle weakness (P2). The concave surface area enclosing the hand trajectory points is found by fitting an alpha surface into the hand trajectory data as described in the previous section. The polygon of the alpha surface defines a boundary which is used to segment the 3D spherical surface. The circumscribed circles of radius π/4 were used to determine the level of concavity.

**Figure 4:**
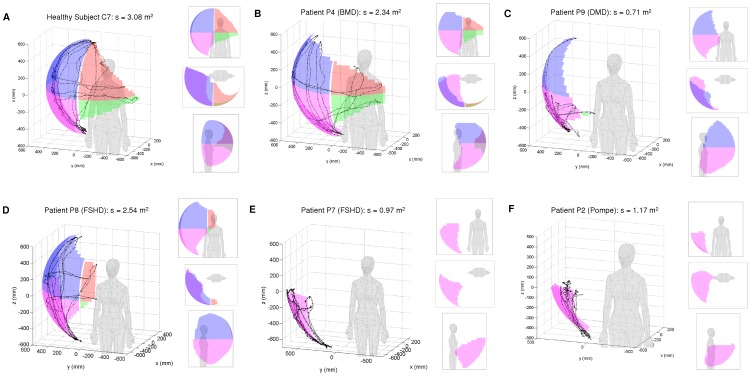
Three-dimensional hand trajectory is projected to spherical coordinates and a spherical surface is fitted to obtain the reachable workspace envelope in 3D space. The surface area is divided and analyzed for each of the four quadrants. Different projections are presented for each subject. The figure shows results in a healthy subject (A), the patients with the following diagnoses: (B) Becker Muscular Dystrophy (BMD), (C) Duchene Muscular Dystrophy (DMD), (D) mild and (E) severe of Facioscapulohumeral muscular dystrophy (FSHD), and (F) advanced Pompe Disease.

Figure 4 shows representative results as obtained in a healthy subject (figure 4A) and various patients (figure 4B–F), showing their 3D hand trajectory with fitted 3D surface. The 3D surface area is divided and analyzed for each of the four quadrants (represented by differently colored patterns for easier visualization). Several representative views are presented for each result. Note that the human model is for illustration only and is not scaled to the actual subject height. The control data of the healthy subject (C7) has a quite equal distribution of surface area between the top and bottom quadrants. The patient with Becker Muscular Dystrophy (BMD), P4, produced similar movement with somewhat reduced reachability at the top of the quadrants. The patient with Duchene Muscular Dystrophy (DMD) was able to perform movement primarily in the lateral coronal and sagittal planes but lacked the strength to raise the arm in the other directions. The results of the two patients with Facioscapulohumeral muscular dystrophy (FSHD) represent the wide range of performance of patients with the shoulder weakness. The patient P8 was able to move into all four quadrants, while the patient P7 only produced movement in the lower ipsilateral quadrant due to the muscular weakness. Finally, the patient with relatively advanced Pompe disease was also able to move his hand only in the lower ipsilateral quadrant resulting in small overall surface area. The difference in 3D reachable workspace and abstracted upper limb functional status can be readily visualized between a healthy individual and individuals with varying degrees of shoulder girdle muscle weakness due to neuromuscular disorders.

**Figure 5:**
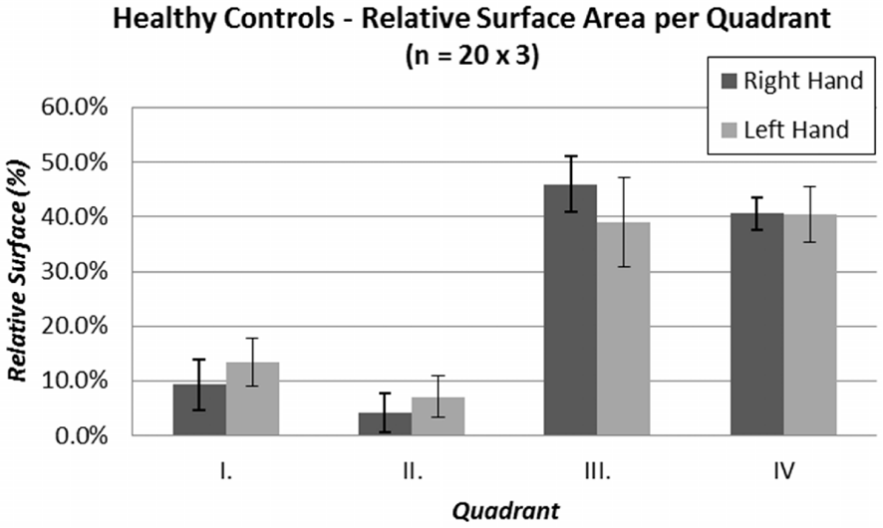
Relative contribution of each quadrant surface to the overall surface area of the reachable workspace envelope as assessed in the group of healthy controls.

Figure 5 shows the mean percentage of the surface area per each quadrant as measured in the group of healthy controls (n = 20×3 trials for each side). The least variability is seen in the quadrant IV as compared to the other three. We believe larger variability in the remaining quadrant is due to the seated position which for some subjects interfered with the cross-body movement (135-degree vertical sweep). Table 2 shows the corresponding absolute and relative workspace area for the group of control subjects as a whole and based on their gender. Due to different arm lengths between males and females, there is greater variability when analyzing the absolute surface area (right side: female 11.6%, male 26.0%; left side: female 17.8%, male: 20.0%). When normalizing the data, the variability within the group is reduced (right side: female 10.4%, male 16.9%; left side: female 15.9%, male: 18.8%) allowing for comparison regardless of the arm length. In the remainder of the paper we present results for the relative surface area that allows for comparison between individuals.

**Table 2:** Data of healthy subjects and their corresponding mean and standard deviation results of the surface envelope assessment are presented for the gender based grouping and as combined total.

	Average		Arm	Quad				Surface	
Group	Age	Side	Length	I	II	III	IV	Relative	Absolute
	(SD)		(cm)	(%)	(%)	(%)	(%)	(−)	(m^2^)
**Females**	41.3	**Right**	71.4	10.4	4.8	44.3	40.5	0.62	1.99
N = 12	(16.0)		(2.8)	(4.2)	(2.7)	(3.3)	(2.3)	(0.06)	(0.23)
		**Left**	71.4	14.4	7.5	38.4	39.6	0.56	1.79
			(2.9)	(3.3)	(3.1)	(5.8)	(2.3)	(0.09)	(0.32)
**Males**	30.3	**Right**	75.0	7.7	3.1	48.4	40.7	0.61	2.19
N = 8	(5.9)		(5.8)	(5.2)	(4.4)	(6.5)	(4.1)	(0.10)	(0.57)
		**Left**	74.8	12.0	6.4	39.9	41.6	0.54	1.91
			(5.7)	(5.5)	(4.9)	(11.3)	(7.7)	(0.10)	(0.38)
**Combined**	36.6	**Right**	72.9	9.3	4.2	46.0	40.6	0.62	2.07
**C1–C20**	(13.6)		(4.5)	(4.7)	(3.5)	(5.1)	(3.0)	(0.08)	(0.40)
N = 20		**Left**	72.8	13.4	7.1	39.0	40.4	0.55	1.84
			(4.4)	(4.3)	(3.8)	(3.8)	(5.1)	0.09	(0.34)

**Figure 6:**
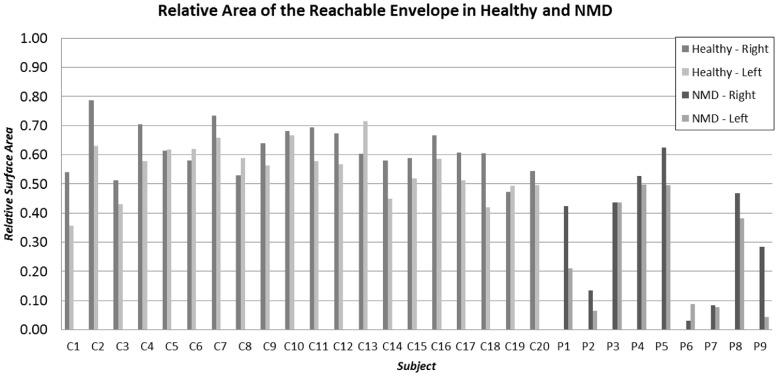
Relative surface area of the reachable workspace envelope as measured in the healthy controls and patients with different neuromuscular diseases (See **Tables 1** and **2** for details and corresponding absolute values).

Figure 6 shows relative surface areas of the reachable envelope in the healthy controls and individuals with various neuromuscular diseases resulting in upper limb weakness. The relative surface area represents the portion of the unit hemi-sphere that was covered by the hand movement. It is determined by dividing the area by the factor 2πr^2^, where *r* represents the distance between the shoulder and fingertips (arm lengths in Tables 1 and 2). This allows scaling of the data by each person’s arm length to allow normalization for comparison between subjects. The subjects covered relative surface area of about 0.60 which corresponds to 60% of the surface area of the frontal hemi-sphere. The mean relative surface area in healthy persons was 0.618 (SD ±0.080) for the right arm and 0.552 (SD ±0.092) for the left arm. See Table 1 for analysis based on gender. An analysis of variance in healthy controls showed that the effect of gender and tested side were not significant factors (*F*_4,36_ = 1.38, *p* = 0.265). Since the patients exhibited quite variable results (and since patients were purposely chosen to represent a wide range of disability and to test the range of detectability for the system), we did not analyze for the group’s aggregate mean value. For the relative surface area results, we performed one-sample t-test to determine if the difference against healthy controls was significant. All patients, except patient P5 (*t*_19_ = −0.6618, *p* = 0.5161) showed significant difference from the control population. In majority of the patients there was no substantial difference between the left and right side, except in patients P1 (Becker muscular dystrophy) and P9 (Duchene muscular dystrophy). For reference, Tables 1 and 2 also include the absolute surface area for each subject.

## Discussion

In this study, we present a novel method to assess the three-dimensional (3D) upper limb functional capacity through quantitative measurement of reachable workspace using a single stereo-camera system combined with customized algorithm. This paper represents development of a portable 3D upper extremity reachable workspace analysis system utilizing a commercially-available stereo camera in conjunction with a customized visualization and analysis program. The accuracy and reliability of the system with its customized acquisition and analysis software algorithm were evaluated against a motion capture system. A comparison of the two demonstrates that the stereo camera system, given the expected trade-offs between accuracy vs. convenience, portability, and cost-effectiveness, is appropriate to capture the 3D hand trajectory. The developed stereo camera-based system was robust, with minimal loss of data points, yet simple to use, and with the average hand trajectory error of about 40 mm, which results to about 5% error of the total arm distance.

At this time, there is no practical and simple 3D acquisition and analysis tool for an upper extremity workspace functional evaluation that is suitable for clinical settings. Traditional upper limb analyses primarily rely on range of motion (ROM) and strength data from segmental regions of the upper limb. Although useful for tracking respective segmental upper extremity function, individual joint range of motion and strength measurements fail to provide a readily visualized overall picture of upper extremity functional capacity. Furthermore, especially for those individuals with significant muscle weakness or contractures as can be often seen in various neuromuscular disorders, individual joint ROM and strength measurements do not adequately portray the extent of upper limb functional deficits.

There are also numerous timed-performance tests and task-oriented functional evaluation methods available for upper extremity, and some have been specifically designed for particular purpose and disease processes. Timed upper extremity functional performance tests such as nine-hole peg test and Jebsen-Taylor hand function test in particular are being considered for clinical application in neuromuscular patients; however, these are limited in that they are restricted to standardized positions/movements prescribed by each test while providing only a limited information about the time (in seconds) to complete a given task. Development of a practical and simple 3D acquisition and analysis tool for an upper extremity reachable workspace functional evaluation would complement the currently available functional and timed-performance tests. Additionally, it would provide a relatively simple, quick, and practical means to compare and track various different upper extremity disorders (various neuromuscular as well as neurological conditions) in an objective and uniform way.

Initially, we investigated quantification of the workspace using volume of the reached space; however, preliminary analysis showed that the volume calculation from the point cloud of the motion data is not robust to outliers. Since the protocol entails movement only for the area away from the body with maximal arm extension, the surface of the workspace envelope turned out to be more representative of individual's performance. Evaluation of upper limb function through surface envelope area of the reachable workspace is a relatively novel area of investigation in a clinical setting. The workspace analysis test aims to measure the envelope of the reachable workspace which will provide information on an individual’s functional abilities. The workspace envelope is defined as the outer surface area of the reachable workspace. The term originates in ergonomics where the maximum workspace data was first reported for an airplane pilot’s workspace [15]. Much effort has been put into evaluation of workspace envelope based on individual’s anthropometric data and the underlying kinematics model of the upper extremity (e.g. [16]–[18]). Such approach is being used for design and development of workstations and other manual equipment in healthy population. In populations with various upper extremity disabilities, increased joint limitations, tendon and muscle injuries, reduced muscle strength, and other neuromuscular conditions, active range of motion (ROM) can significantly affect the reachable workspace envelope. Klopčar and Lenarčič [6] presented a method of determining a reachable workspace based on developed kinematics model of the arm and standardized joint range of motion measurements performed in physiotherapy. However, these can be time and resource intensive efforts, and have not yet found utility in clinical settings. The results from this study demonstrate, for the first time, that it is feasible to apply the reachable workspace surface envelope area concept to data obtained using a single stereo-camera system in a simple and practical manner suitable for various clinical purposes.

The application of the developed 3D workspace acquisition system using a stereo-camera and a customized algorithm to determine the surface envelope area was demonstrated on actual individual patients with varying degrees of upper limb dysfunction due to neuromuscular diseases (Becker muscular dystrophy, Duchenne muscular dystrophy, Facioscapulohumeral muscular dystrophy and Pompe disease). The results have been promising and suggest that the developed methodology has adequate range of sensitivity to determine not only the healthy individuals from those with neuromuscular disorders, but also capable of separating out those with severe upper limb dysfunction from those with milder phenotypes. In patients with neuromuscular diseases there is a substantial need for quantitative assessment methods which could track progress of the disease or effects of novel treatment methods [19]. Many of the functional tests are not specific enough for wide range of impairments resulting from neuromuscular diseases and they provide only qualitative assessment. Even in goniometric measurements of range of motion, wide variation in inter-tester reliability has been found in children with Duchene Muscular Dystrophy [20]. Our initial results suggest that the evaluation through reachable workspace envelope can provide quantitative information on ability to reach for objects with straightforward at-a-glance visualization of the overall functional capability of the upper limb. Our results also suggest that similar methodology can be applied towards post-surgical patients as well as tracking therapeutic efficacy during physical therapy and pharmacologic treatments (in clinical setting and drug trials).

Although only a few patients were examined in this pilot study and further studies are needed to define the loss of workspace surface area in neuromuscular diseases, an interesting observation was noted when analyzing the pattern of loss of function in terms of quadrant area reduction in patients with neuromuscular disorders ranging from mild to severe phenotypes. It makes sense intuitively and it appears true, that as the disease progresses, the upper quadrant function is lost. Following that, the results indicate loss of medial bottom quadrant, with the lateral bottom quadrant function being the most preserved into the late stages of the neuromuscular diseases that were examined. This pattern of loss of function may correlate with loss of self-care capabilities and activities of daily living (ADL: personal hygiene and grooming, feeding, dressing, toileting), and may represent a very interesting area of future research.

The primary weakness of the system is that it still relies on the visibility of the markers, their accurate detection and reliable tracking. In our initial experiments we have found that it is difficult to apply standard tracking methods (e.g. predicting smooth movement trajectories) as many of the patients produced rather abrupt trajectories. The markers attached to clothing or skin also exhibit movement that can affect the accuracy in joint movement assessment. Several post-processing steps described in the Methods section were applied to remove the outliers created due to errors in tracking. The tracking errors likely contributed also to the larger variability of the data between the healthy controls. Another variable that we did not fully control for was the interpretation of the movement patterns by different individuals. The most variability was seen for the movement across the body since some subjects were trying to avoid occlusion with their legs in the seated position. Another factor affecting the relative surface area division may stem from the fact that we have used subject's initial (neutral) pose as the reference for the determination of coordinate systems. Since the neutral pose was not fully consistent between subjects, it may have increased the variability of the data.

The system in its current form would not be able to detect ‘trick’-movements (or compensatory maneuvers) of the upper extremity where individuals compensate for the loss of function in certain muscle groups by utilizing other muscles and body parts. For example, a person could lean forward to increase his/her range of motion. We are planning to more closely analyze the trick movements in individuals with neuromuscular diseases in our future studies. The algorithm will need to more accurately track the location of other body parts such as the trunk, shoulders and elbow during the execution of the protocol. Detecting for example rotation of the trunk would indicate that the subject is using a compensatory-movement to increase the range of reaching. We are currently evaluating and developing marker-less body tracking methods to track the trajectories of the upper limbs over time using stereo cameras or active depth cameras [21]. We are also investigating how to define the movement trajectories more accurately through visual feedback on the computer screen or physical markers in the environment.

Future studies will need to validate the reachable workspace envelope area against other validated measures of upper extremity function. Possible studies may involve comparison and validation studies involving the various timed performance tests, task-oriented tests, and strength measures. Potentially in the future, other clinically valuable information such as quality of movement can also be provided by similar vision-based motion tracking systems. Other potential area of clinical application of the developed system may be in tele-medicine and tele-rehabilitation realms.

## References

[1] Klopčar N, Tomsic M, Lenarčič J (2007) A kinematic model of the shoulder complex to evaluate the arm-reachable workspace. J Biomech 40: 86–91. 16387308 10.1016/j.jbiomech.2005.11.010

[2] Schiele A, Helm van der FCT (2006) Kinematic design to improve ergonomics in human machine interaction. IEEE Trans Neural Syst Rehabil Eng 14: 456–496. 17190037 10.1109/TNSRE.2006.881565

[3] Gajdosik RL, Bohannon RW (1987) Clinical measurement of range of motion. Review of goniometry emphasizing reliability and validity. Phys Ther 67: 1867–1872. 3685114 10.1093/ptj/67.12.1867

[4] Kolber MJ, Fuller C, Marshall J, Wright A, Hanney WJ (2012) The reliability and concurrent validity of scapular plane shoulder elevation measurements using a digital inclinometer and goniometer, Physiother Theory Pract. 28: 161–168. 10.3109/09593985.2011.574203 21721999

[5] Mullaney MJ, McHugh MP, Johnson CP, Tyler TF (2010) Reliability of shoulder range of motion comparing a goniometer to a digital level. Physiother Theory Pract 26: 327–333. 20557263 10.3109/09593980903094230

[6] Klopčar N, Lenarčič J (2005) Kinematic model for determination of human arm reachable workspace. Meccanica 40: 203–219. 10.1016/j.jbiomech.2005.11.010 16387308

[7] Schmidt R, Disselhorst-Klug C, Silny J, Rau G (1999) A marker-based measurement procedure for unconstrained wrist and elbow motions. J Biomech 32: 615–621. 10332626 10.1016/s0021-9290(99)00036-6

[8] Roux E, Bouilland S, Bouttens D, Istas D, Godillon-Maquinghen A-P, Lepoutre F-X (2001) Evaluation of the kinematics of the shoulder and of the upper limb. In: Proceedings of the 3rd Conference of the International Shoulder Group. Delft University Press, 66–71.

[9] Roy JS, Moffet H, McFadyen BJ, Macdermid JC (2010) The kinematics of upper extremity reaching: A reliability study on people with and without shoulder impingement syndrome. Sports Med Arthrosc Rehabil Ther & Technol 2: 1–12. 20331889 10.1186/1758-2555-2-8 PMC2857852

[10] Veeraraghavan A, Srinivasan M, Chellappa R, Baird E, Lamont R (2006) Motion based correspondence for 3D tracking of multiple dim objects. In: Proceedings of the International Conference on Acoustics, Speech and Signal Processing (ICASSP). IEEE Press, 669–672.

[11] Zheng J, Zhou B, Li X, Yan J (2008) Multi-markers 3D tracking algorithm in a video motion capture system. In: Proceedings of 5th International Conference on Visual Information Engineering (VIE 2008), SEMEDIA, 52–55.

[12] Mori N, Suzuki T, Kakuno S (2007) Noise of acoustic doppler velocimeter data in bubbly flows. Journal of Engineering Mechanics 133: 122–125.

[13] Sengupta AK (1998) A model of three dimensional maximum reach envelope based on structural anthropometric measurements, In: Kumar S, editor. Advances in Occupational Ergonomics and Safety. Ohio: IOS Press, 256–259.

[14] Bernardini F, Bajaj C (1997) Sampling and reconstructing manifolds using alpha-shapes. Report Number: 97-013. West Lafayette(IN): Dept Comput Sci, Purdue Univ.

[15] Kennedy KW (1978) Reach capability of men and women: A three dimensional analysis. Report No. AMRL-tr-77–50. Wright-Patterson Air Force Base, OH: Aerospace Medical Research Laboratories.

[16] Li S, Xi Z (1999) The measurement of functional arm reach envelopes for young Chinese males. Ergonomics 33: 967–978.

[17] Abdel-Malek K, Yang J, Brand R, Tanbour E (2004) Towards understanding the workspace of human limbs. Ergonomics 47: 1386–1405. 15513715 10.1080/00140130410001724255

[18] Behara DN, Das B (2010) Anthropometric modelling for the determination of 3-D maximum functional reach. Theoretical Issues in Ergonomics Science 12: 87–101.

[19] Zupan A (1996) Assessment of the functional abilities of the upper limbs in patients with neuromuscular diseases. Disabil Rehabil 18: 69–75. 8869508 10.3109/09638289609166020

[20] Pandya S, Florence JM, King WM, Robison JD, Oxman M, et al (1985) Reliability of goniometric measurements in patients with Duchenne muscular dystrophy. Phys Ther 65: 1339–1342. 4034668 10.1093/ptj/65.9.1339

[21] Obdržálek S, Kurillo G, Han J, Abresch T, Bajcsy R (2012) Real-time human pose detection and tracking for tele-rehabilitation in virtual reality. Stud Health Technol Inform 173: 320–324. 22357010

